# The Challenges of Using Reclaimed Asphalt Pavement for New Asphalt Mixtures: A Review

**DOI:** 10.3390/ma13184052

**Published:** 2020-09-12

**Authors:** Giulia Tarsi, Piergiorgio Tataranni, Cesare Sangiorgi

**Affiliations:** Department of Civil, Chemical, Environmental and Materials Engineering–University of Bologna, 40131 Bologna, Italy; giulia.tarsi2@unibo.it (G.T.); piergiorg.tataranni2@unibo.it (P.T.)

**Keywords:** recycling, reclaimed asphalt pavement, wasted asphalt, recycled asphalt mixtures

## Abstract

Reclaimed Asphalt Pavement (RAP) material mainly consists of removed asphalt concretes from existing infrastructures and, to a minor extent, of wasted or rejected mixes during the production processes. Being composed of two valuable non-renewable resources, i.e., aggregates and bituminous binder, its conscious use can ensure the sustainability of asphalt pavement construction. Thanks to the use of RAP material in new asphalt products, the USA saved 4.1 million tons of virgin binder and 78 million tons of virgin aggregates in 2018. Therefore, the use of RAP for the production of new asphalt formulations at the top of the recycling hierarchy is preferable instead of being down-cycled in low-value applications. The RAP material represents one of the most re-used construction products worldwide; in 2018, approximately 88% wt. and 72% wt. of RAP were used in USA and Europe, respectively, as aggregates for Hot, Warm and Cold Asphalt Mixtures and for unbound layers. Several studies have revealed positive responses of the recycled asphalt mixtures with high or very high content of RAP. However, the common practices of many countries still limit the RAP content to a 15–20% wt., on average, in the recycled asphalt mixes. The amount of RAP in asphalt concretes can be significantly increased by applying good management practices of the RAP, either processed or not, as well as novel production technologies and advanced mix design approaches. This manuscript aims to summarize the state-of-the-art of use of RAP aggregates in new asphalt mixtures. The economic and environmental benefits are also discussed.

## 1. Introduction

The asphalt concrete removed from an existing road pavement is a fully recyclable material for construction [1,2]. Since it consists of valuable non-renewable resources, i.e., approximately 95% wt. of aggregates and 5% wt. of aged bituminous binder, it can be re-used in new asphalt mixtures, reducing the demand for virgin aggregates and bitumen, or as recycled aggregates to produce unbound layers of pavements [3]. However, the latter option does not exploit the full potential of the material as the former does, since it ignores the aged bituminous binder that coats the recycled aggregates. The value of removed asphalt concrete is maximized by re-using it in the same engineering applications at the top of the recycling hierarchy and not in being down-cycled as simple aggregates [3]. Eventually, the downgrading of the removed bituminous mixture into a lower value product (i.e., aggregates for unbound layer) may be appropriate after several re-uses and recycling processes [1]. Indeed, some studies have revealed the possible multi-recycling aspect of the removed asphalt concrete [4,5], that is, the capacity of the material in being treated with multiple recycling processes without losing its properties and performing some valorisation actions when required [6]. The recycle and/or the re-use of removed bituminous mixture is an example of the sustainable development in the infrastructures sector. The encouragement of its use reflects the worldwide trend to face the existing environmental issues by trying to increase the efficient use of resources and reduce carbon emission [7]. Nevertheless, the use of removed asphalt concrete has to be evaluated also from a cost efficiency perspective, since it significantly reduces the overall costs of new bituminous products [2,8].

The wasted asphalt mixtures during the start-up, transition between mixes and clean-out operations together with the rejected mix from a project have to be accounted for in the total amount of recyclable bituminous materials, adding it to the major quantity of the removed asphalt concrete from existing pavements. Generally, standards and guidelines that regulate the handling and the inclusion of the waste asphalt material in other bituminous products consider both origins. In detail, the EN 13108-8 standard defines the Reclaimed Asphalt (RA) as the processed material in the form of milled or the ripped up slabs from existing bituminous road layers and the asphalt mixtures from surplus, rejected or failed productions [9]. In other countries, notably in the USA and Australia, the removed asphalt materials for road pavements are known as Reclaimed Asphalt Pavement (RAP) to differentiate it from other reclaimed materials that incorporate bituminous binder, e.g. shingles [3]. Hereafter, the reclaimed and wasted asphalt will be indicated as RAP, although both acronyms are widely used. Most of the guidelines and countries or state regulations, such as the European one (EN 13108-8), specify that RAP should be a ready-to-use constituent material for producing new bituminous mixes. This statement may involve some additional operations, such as crushing, grading, fractionating and homogenization processes after the removal of the asphalt concrete. Moreover, all operations can be performed under various degrees of complexity.

Four different techniques can be adopted to remove the existing bituminous layers of pavements and to recycle the obtained material, which are divided based on the energy input of the recycling process and the place where the RAP is used for the production of new materials. The former category can involve the heating of any materials, while the latter can imply the transportation of the materials. Therefore, the possible recycling techniques are: cold in-place recycling, hot in-place recycling, hot in-plant recycling and full depth reclamation [10]. This review will focus on hot in-plant recycling of asphalt mixtures containing RAP to represent a complete and extensive overview of the use of RAP, highlighting the related benefit and possible constraints.

## 2. An Overview of The RAP Usage Worldwide

The percentage of re-used RAP has grown gradually in many countries around the world [3]. Currently, a considerable quantity of this recycled material is used for the production of Hot Mix Asphalt (HMA) and Warm Mix Asphalt (WMA) mixes, which represent the ideal re-use of RAP aggregates as previously mentioned. Nowadays, according to several studies, a RAP content in the range of 15–20% wt. is becoming a standard practice for the production of bituminous mixtures.

The introduction of RAP in the new formulations of asphalt concrete up to 40% by the total weight of the mix has been conducted in South Africa since 2009 [3]. A decade later, in-plant recycling up to 40% wt. of RAP content has become a common industrial practice [3].

The Japanese road sector was an example of an efficient recycling system of RAP already in 2013. Two years later, 99% wt. of the total amount of RAP was re-used [11]. A small quantity of RAP was employed in base course layers, while the majority was added in new HMA–WMA mixtures, introducing about 47% wt. of RAP on average [11]. The relatively small area of Japan, with restricted raw material resources and limited space for waste disposal, has motivated the search for sustainable solutions able to maximize the conservation of natural resources and minimize waste [11].

Similarly to the Japanese trend, the latest yearly survey of the National Asphalt Pavement Association (NAPA) highlighted that the USA asphalt industry re-used more than 99% wt. of the available RAP in 2018, which has represented the most recycled product among American states [8]. The estimated results have been obtained thanks to the participation of 49 states, two USA territories and the District of Columbia [8]. In detail, approximately, 97% of the re-used RAP was recycled in bituminous products, mainly HMA and WMA mixtures, while 3% was re-used in other civil engineering applications. As a consequence, the average quantity of RAP landfilled was almost zero in 2018; the producers reported landfilling about 12 thousand tons of RAP that is about 0.012% [8].

The European Asphalt Pavement Association (EAPA) monitors all European activities related to asphalt, including the re-use and/or recycling of RAP. Thanks to the data provided by members from each country, the average percentages of RAP used in 2018 were determined; when data from some countries were not available were marked as “no data” and the quantity was accounted as zero. On average, the European nations mainly used RAP in HMA and WMA mixtures productions and other asphalt products, i.e., Half Warm Mix Asphalt and Cold Mix Asphalt (CMA) mixtures. However, a remarkable percentage of RAP, higher than 20% wt., was down-cycled as aggregates for unbound layers, and 8.4% wt. of the available recycled asphalt materials was landfilled in 2018 [12]. These values represent the average quantity of 14 countries, where some virtuous nations compensate the common practice of others that preferably use the RAP aggregates in unbound layers. It should be pointed out that the European data are very variable from country to country and the use of RAP is highly dependent on national regulations.

In Table 1, the rough percentages of the RAP statistical average uses in the USA and Europe are compared. The listed values were deduced considering the estimated data by the American survey of RAP uses in 2018 [8] and the corresponding quantity of RAP that has been accepted by producers and the mean European results of the collected data accounting as zero when ‘no data’ are available [12], respectively. Among all RAP uses, the American and European common practices mainly differ in the quantity of RAP that is introduced in new HMA/WMA mixes and the quantity used as aggregates for unbound layers. Furthermore, the introduction of RAP in the CMA formulations of American and European nations varies by more than 3 percentage points. This difference highlights a dissimilar trend adopted by the two groups of nations. However, the European data of the RAP used in CMA mixes reflect the large use of RAP for this scope by a limited number of nations; the majority of EU nations do not introduce RAP in CMA mixtures or data are not available. In total, Europe disposes a very high quantity of RAP compared to the USA. Hence, the percentages listed in Table 1 highlight that the European states could exploit more the recycled aggregates potential.

Although the values in Table 1 show a high recycling rate of RAP, there is a surplus of unused recycled asphalt concrete in both areas because more RAP was received than used [8,12]. The widely spread perception that recycled materials have lower quality than new products, together with the various and negative concerns related to the RAP use, led to a lack of confidence in using high contents of RAP for the production of new bituminous mixtures [2,13]. These misconceptions turn in restrictive national or regional regulations that limit the re-use of RAP in the new asphalt mixtures. Hence, the restrictions cause an increase in the RAP stockpile inventory [14]. Reporting some examples, the majority of state road agencies in the USA and Canada allow the use of RAP content higher than 15% wt. in dense graded asphalt surfacing layers. However, the number of agencies drops to 5 when the acceptable RAP content reaches and/or exceeds 30% by the total weight of the mix [15]. The effective RAP content introduced in HMA–WMA mixes was about 21.1% wt. in the USA; among all the states, only 19 states added more than 21% wt. of RAP, on average, in their asphalt products [8]. However, the average quantity of RAP introduced in the new HMA and WMA mixtures is less than the usual percentage used in South Africa and Japan. The Australian states of Victoria, New South Wales and Tasmania and New Zealand allow a RAP content of at least 15% wt., with the possibility to reach higher quantities if the contractors demonstrate that they adopt suitable plant technologies and quality control procedures [15]. Conversely, the states of Queensland, South Australia and Western Australia do not permit the use of RAP for road surface layers [15].

## 3. Limits to the Increase of RAP Content in HMA Mixtures

In 2011, the Federal Highway Administration (FHWA) defined the asphalt concretes containing more than 25% of RAP by total weight of the mix as high-content RAP mixtures [16]. Several studies have demonstrated the feasibility of producing bituminous mixtures with high (over 40% wt.) or very high (up to 100% wt.) RAP. The negative perceptions and the practical issues that limit the common practice to go beyond the average RAP content of 15–30% wt. in HMA mixtures can be summarized in four categories that refer to the quality of the RAP aggregates, the technology of the production plant, the mix design methodology and the performances of the final mix containing RAP.

### 3.1. Quality and Homogeneity of RAP Aggregates

In order to consider the RAP material as a controlled input for asphalt mix design, the quality and homogeneity of the RAP aggregates and stockpiles should be assessed [15]. Generally, the removed asphalt concrete presents a high content of fine particles, due to the milling operations of the pavement layers and/or to the crushing processes to reduce the aggregates size, especially when the material comes from a full-depth pavement demolition [10,16]. The amount of fine particles may limit the maximum RAP content because the gradation requirements of the final mix might not be met [16]. Furthermore, the finer particles contain higher amount of aged binder, due to their higher surface area [17]. Still, finer particles tend to retain moisture [10,13]; the RAP material itself does not drain as virgin aggregates do [10,16]. All these aspects must be taken into account for correct RAP management, mix design and production of asphalt concretes containing RAP. Milled aggregates from a single and traceable source can be very consistent and, sometimes, no further crushing and/or screening operations are needed to use the material in new asphalt concretes [16]. However, the quality of the removed asphalt materials from pavements depends on the milling machine, its speed and milling depth; in particular, the latter factor is important as the material from a single layer has homogeneous properties (i.e., aggregate type and grading curve, bitumen characteristics and content) [16,18]. A good milling operation guarantees a high quality of the removed asphalt [18]. As a result, keeping millings of specific projects in separate stockpiles and ensuring that the removed asphalt concrete is not contaminated with a bituminous base layer can increase the consistency of the waste asphalt material [16]. Despite the advantages, it is often impractical for asphalt companies to store the material from different sources in separate piles, before and after their processing, due to the limited area of the production plant and to the generally limited amount of asphalt coming from a single project [19,20]. In these cases, additional operations should be performed to increase the homogeneity of the material before its characterization and use in new asphalt mixtures. A detailed discussion about the best practices for the management of removed asphalt concrete and the characterization of RAP material will be further addressed in this paper.

### 3.2. Current Plant Production Technologies

The RAP material cannot be incorporated in standard plants as virgin aggregates because excessive blue smoke will be produced if the recycled material comes into contact with the burner flame due to the combustion of the aged binder [10]. Most of the traditional production technologies limit the maximum amount of RAP that can be added in the new asphalt products [14]. The restriction of RAP content in new formulations produced in traditional batch plants is imposed by the production process, which usually introduces the RAP material at ambient temperature by overheating the virgin aggregates, which leads to the drying and heating of the RAP material by conduction when the aggregates come in contact [10,14,19]. The cold and damp RAP aggregates, which commonly contain 7–8% of moisture [21], lead to a decrease in the production rate as they may require an extended mixing time to adequately be dried and heated [13]. In addition, the maximum RAP content depends on the allowable moisture percentage in the final asphalt mixture [10]. During the contact between the superheated virgin aggregates and the cold RAP material, important heat loss is usually experienced [10,13]. In addition, the mixing operation of aggregates leads to release and build-up fines and binder on various parts of both type of plants [10,13]. A practical issue of batch plants related to the inclusion of cold recycled aggregates is the generation of steam when they come in contact with the superheated virgin materials; the combination of the two materials will cause a mild explosion and emission released by moisture and dust [10,22]. For these reasons, it is always recommended to keep the RAP aggregates as dry as possible. Thanks to some modifications of the traditional batch plants, the current technology allows the incorporation of 30–35% wt. of RAP [10]. In order to include RAP contents above this limit (30–35% wt.) in the batch plants and limit the abovementioned issues, it is necessary to warm the recycled aggregates. In drum plants, the RAP material is commonly heated, allowing the introduction of recycled materials up to 50% wt. [10]. Various equipment and technologies can be adopted to warm RAP aggregates preliminary and indirectly in both type of production plants. Indeed, direct contact between RAP material and the flame should be avoided in all plants. The prolonged exposure of RAP aggregates with superheated virgin material should be also limited. Both operations lead to further aging the RAP binder and to the release of hydrocarbons emissions (blue smoke) [10,22]. On the other hand, the correct pre-warming time of RAP material has to allow the particles to sufficiently soften, break down and blend with the virgin materials [23,24]. The technical issues related to the RAP content limitations can be overcome thanks to the upgrading and/or modification of most of the available asphalt plants [19]. However, the additional costs of plant upgrading and/or modification should be assessed [6]. The modes for incorporating RAP aggregates and the advances in production plant technology that are able to cope with the abovementioned issues will be further discussed in the following sections.

### 3.3. Undefined Mix Design Method for Binders and Mixes

The mix design of asphalt mixtures that incorporate RAP aims to meet the same performance criteria as asphalt concretes containing virgin aggregates only [15,21,25,26]. However, the traditional mix design methodology has to be modified for mixtures with high and very high-contents of RAP in order to take into account the characteristics of this material in terms of aggregates gradation and aged binder properties and content. The management of RAP aggregates is strictly related to the mix design as the homogeneity of the RAP material allows the characterization of each RAP stockpile to be consistent. Hence, designers have to consider the recycling processes of the RAP aggregates and identify which size fractions of RAP stockpiles best satisfy the required mixture gradation, binder content, mixture volumetric and performance requirements, while optimizing the use of the available material [14]. The RAP binder contributes to the rheological properties and the total content of the final blended binder (i.e., aged and virgin binders) and of the final asphalt mix. Most of road state agencies and country regulations assume a full blending between the aged and virgin binders [2,16,27]. Conversely to this widespread practice, according to several studies, only a portion of the RAP binder is available and can act as a binder in the new formulation [28,29]. The definition of the degree of blending and the diffusion that will occur between the virgin and aged binders represents one of the main obstacles to the design asphalt mixtures with high and very high contents of RAP, as there are no overall well-defined procedures for their determination [19,28]. Furthermore, increasing the RAP content may require the addition of softening agents and/or rejuvenators to restore the properties of the aged binder [29]. The introduction of any recycling agent represents an additional variable in the mix design, requiring the selection of a compatible agent and its dosage. Unfortunately, the blending of aged binder with recycling agent is still under study [28]. A comparison of the standard practices and the latest proposed mix design for mixtures with high and very high-contents of RAP are discussed in the paper.

### 3.4. Performances of the Resulting Asphal Mixtures

From a mechanical point-of-view, the increase of RAP content in new formulations implies a potential increase in the stiffness of the final asphalt mixes [16,25], which is mainly dependent on the stiffness of the RAP binder [29]. A stiffer asphalt concrete might be less resistant against cracking due to fatigue, thermal shock and cracking reflection, representing the main reason for the reluctance of road agencies to the use of RAP mixtures [25]. Several studies highlighted that the presence of aged binder in well-designed asphalt concretes allows the resulting mix to be less susceptible to water damage and less prone to permanent deformations [19,27,30]. However, many factors may negatively affect the water sensitivity of the final asphalt mixture, such as insufficient blending between aged and virgin binders during the in-plant production [31] and the stripping tendency of the milled pavement from which the recycled aggregates originate [32]. As for the permanent deformations, the use of a softer virgin binder and recycling agent might influence the rutting resistance of the final asphalt concrete [25,27]. Furthermore, the use of incompatible agents or excessive dosages of additives could lead to flushing phenomena, such as the migration of binder towards the surface of the bituminous layer [14]. Hence, during the mix design of high and very high content RAP mixtures, the use of softer binder and/or recycling agent should be balanced in order to mitigate the increased stiffness, without over-softening the resulting binder, to increase the rutting potential [16,33]. The performances of virgin and high and/or very high content RAP mixtures will be discussed in a section of the present review, with a specific focus on the main associated distresses.

## 4. Optimization of RAP Management

The processing of milled asphalt, full-depth demolition and wasted asphalt plant mixes becomes more and more important as larger RAP content is introduced in new bituminous formulations [3]. The management and quality control operations of RAP represent the starting procedures to ensure consistency and quality of the final products. Hence, this affects the flexibility in RAP utilization.

### 4.1. Towards Recycled Asphalt Materials

Processing of RAP material may involve one or more operations, i.e., checking for contaminants, screening, crushing and fractioning, to create consistent RAP materials that meet standards for bituminous mixtures production [16].

Regardless of the origin of the removed asphalt, the incoming materials should be free from contaminants; the RAP should be inspected to avoid the dumping of soil, construction debris or any deleterious materials [2,3,16,21]. It is recommended to examine the material before it is unloaded on the unprocessed stockpile in the recycling plant [21]. The European regulation, EN 13108-8, underlines the importance of this operation by requesting the evaluation and cataloguing of foreign matters into unprocessed RAP material [9].

The removed asphalt mixture from a single traceable source may not require further processing if correctly milled [16,18]. In these cases, if the maximum aggregate size is suitable to be used in the desired mix design, the classified material from specific projects should be properly stored in arc-shaped stockpiles with multiple layers to minimize segregation and water retention and avoid contamination; then, aggregates undergo sampling and testing to evaluate their properties and homogeneity [16,20]. Avoiding further operations allows the reduction of RAP processing costs. On the other hand, the removed asphalt material will be subjected to screening and crushing processes if the maximum size of aggregates is too large [20]. When RAP from multiple sources could not be separated in different piles, it is necessary to carefully blend the unprocessed asphalt material before any further operations [10,20]. Successively, the RAP aggregates are subjected to the same procedures, screening and, possibly, crushing. The crushing process can improve the consistency of the final RAP material from multiple sources [16,20]; however, this operation usually creates more fine aggregates as mentioned in the previous section. In order to reduce this negative aspect, it is suggested to screen the unprocessed RAP aggregates before they enter the crusher, allowing the finer particles to bypass this operation [20].

Furthermore, the RAP material may be fractionated in similar sized particles to improve the mix design flexibility as it would be beneficial for controlling the quality of the final mix that contain RAP aggregates [13,17,19,20]. This operation is strongly related to the existing and target gradation of the mixture [19]. The fractionation of RAP aggregates has begun to be recommended and required by some road agencies, especially for the production of mixtures with RAP content higher than 20% wt. [13,20,21]. However, the latest survey conducted by NAPA shows that the use of fractionated recycled aggregates does not directly correlate to a higher utilization percentages of RAP material in production plants [8].

A well-managed RAP material from multiple sources, also without a fractionating process, is typically more consistent than virgin aggregates [21]. Indeed, a better approach to assure consistency of RAP stockpile is to set limits on its variability [20]. The main goal of RAP processing is the creation of uniform and consistent stockpiles. Based on the final mix requirements, this aim can be achieved by performing consequent operations:

- Check and remove possible contaminants from unprocessed material;
- Reduce the maximum size of aggregates minimizing the formation of finer particles;
- Possibly execute the RAP fractionation.

It is recommended that asphalt material processing occurs prior to its feeding to the production plant [16]. Moreover, processed asphalt is likely to be moved from the location in which it is screened and/or crushed to the place more in which it is more convenient to be fed into the asphalt plant; this movement represents an opportunity to remix the material and improve its consistency [20].

### 4.2. Best Practices for Stockpiling RAP Aggregates

Segregation, consolidation and moisture retention are the major issues related to the unprocessed and processed RAP stockpiles, which can be prevented and/or limited by an adequate stockpiling of the RAP material. In general, normal practice, as for virgin aggregates stockpiles, should be adopted against segregation and consolidation phenomena; however, moisture retention is a specific issue of RAP aggregates that requires additional attention. RAP stockpile shapes represent the first parameter in the evaluation of the management of the material. From a geometrical point of view, different shapes of the related RAP stockpile are suggested, as reported in Table 2.

Segregation results in a higher amount of coarse aggregates that contain a lower percentage of aged binder at the base of the stockpiles; meanwhile, finer particles with higher aged binder content are at the top of the stockpile [20]. A small bulldozer can form an arc-shaped and layered stockpile of unprocessed RAP material by pushing the aggregates in multiple layers, taking care not to push the material over the slopes, as that will potentially segregate the aggregates themselves [16]. The layered stockpile allows a better consistency of the final RAP aggregates, as the loader will approach the stockpile from the side and dig up through material to process the aggregates from numerous layers [20]. Regardless the origin of the processed RAP (from single or multiple sources either fractionated or not), the stockpiles are preferred to be built in a conical shape or small, low-sloped piles [10,16]. The segregation of RAP material becomes a common problem when stockpiles are built using fixed conveyors that allow the RAP aggregates to drop onto the stockpile; this method leads larger particles to roll down towards the bottom of the stockpile, since they have more kinetic energy [20].

The height of the RAP stockpiles is limited to 9 meters to reduce its potential self-compaction [20]. In addition, the use of small and light bulldozers is advised to drive on the stockpile [16,20].

The tendency of RAP material to hold water leads to higher moisture content in RAP stockpiles than the values generally found for virgin aggregates stockpiles [19]. The percentage of moisture is a key parameter affecting both production rate and drying costs [20], which limit the maximum amount of RAP content in the new asphalt concretes [14]. The use of conical stockpile without surface depressions that facilitate the water retention allows a natural shedding of rain or snow [20]. The crust naturally formed on the superficial 20–25 cm of stockpile helps to shed water as well [10,16]. However, further specific measures can be taken to minimize the accumulation of moisture, such as covering the RAP stockpiles and storing RAP material on a paved surface [10,16,34]. Storing RAP stockpiles under a shelter or building prevents precipitation from wetting the RAP aggregates [20]. This precautionary operation is recommended by several national guidelines on RAP management and especially with regard to the processed materials [2,3]. RAP storage on a solid and sloped surface may facilitate the drainage of water, avoiding moisture infiltration in the subgrade. Furthermore, it may prevent the contamination or the compaction of the underlying surface [10]. Conversely to the American and the South African suggestions, the Australian Asphalt Pavement Association (AAPA) recommends that the RAP stockpile is formed on a sloped pad [2].

### 4.3. Sampling and Characterization of RAP Material

Sampling is an important procedure in order to characterize RAP aggregates. The specimens should be taken from each stockpile involved in the new asphalt concrete production to verify its homogeneity and allow characterization of the RAP [2,16]. Once the RAP material has been processed, the material is moved to a more suitable position to be fed into the hoppers of the plant and it is recommended that RAP stockpile be sampled in this latest position [20]. Sampling and testing requirements vary according to the source of RAP and its content in the new mixture, as reported in American guidelines [16,17]. Furthermore, the standard EN 13108-8 suggests to take into account the final use of the new asphalt product containing RAP to define the frequency of tests [9].

Sampling involves the collection of material from multiple positions around the stockpile, which have to be chosen randomly [15,16,17,20]. Then, a portion of each sample is tested in order to determine the consistency of the RAP stockpile, and the quantity remainder are combined into one representative sample of the stockpile for conducting mixture design [15,16,17]. The FHWA and NAPA associations claim that the best practice for sampling is to perform a set of 5 or 10 (as a minimum value), respectively, but preferably more than 10 tests per 1000 tons of RAP material [15,16]. The European standard (EN 13108-8) also fixes the minimum number of tests as 5; however, it should be performed per 500 tons, 1000 tons or 2000 tons depending on the source of RAP material [9]. The RAP samples from different locations in the considered stockpile are used to calculate the variability statistic and the results showed that RAP material from multiple sources can also be as consistent as the material milled from a single project [20].

The basic characterization of RAP materials involves the determination of RAP aggregates gradation, binder content and the amount of retained moisture. Additional tests, such as RAP aggregates quality testing and aged binder properties testing can be requested by each road agency and regulation, especially if a remarkable amount of RAP is proposed for the final asphalt mix [17]. For instance, the common American practice based on the Superpave method requests the evaluation of RAP aggregate density and the determination of consensus properties (i.e., angularity, flakiness and elongated indexes and clay content) in the basic characterization of the recycled aggregates recovered from RAP materials [17,20]. The properties of the aged and blended binder are required by most of the national road agencies if the RAP content is greater than 25% wt. [17,20]. The South African Association, SABITA, requests additional tests (such as aggregate crushing value, 10% fines aggregate crushing value, flakiness index for aggregates and softening point, penetration and performance grade parameters for recovered RAP binder) when the RAP content exceeds 15% by the total weight of the mix [3].

## 5. Asphalt Production Plant Technologies

**Figure 1:**
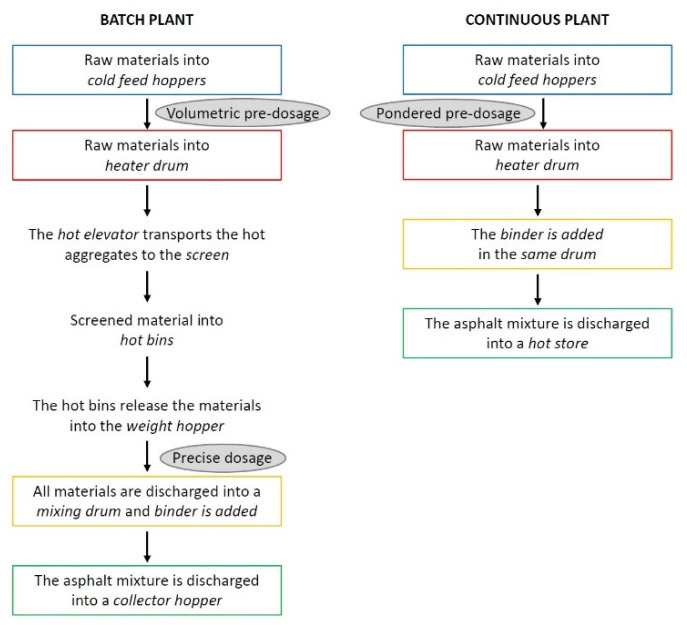
Operational procedures of batch and continuous plants.

HMA mixtures can be produced with two main types of asphalt plants: batch and continuous (drum) plants; both of them allow the production of the same mixture with the same characteristics [22]. They differ in the production process, as the batch plants allow dosing and, then, feeding the mixing drum with each component separately in order to produce a specific quantity of the defined mixture [35]. While, the continuous plants do not provide discontinuity of operations because the components are added in order to ensure a continuous flow of the final asphalt concrete [35]. In Figure 1, the typical operational procedures of both plants are schematised. The batch plant offers more flexibility on the mix design as a variety of aggregate type and dimension can be introduced in the cold feed hoppers [22,35]. Furthermore, during production, aggregates are screened for a second time and weighed after being heated and dried ensuring that their moisture content does not affect the weight of the material [35]. Hence, in a batch plant, the constituent materials undergo to a larger number of screening and checking operations. On the other hand, the continuous plant has a higher rate of production and, being a simpler system than batch plant, it allows the reduction of maintenance costs [22,35]. However, it has the disadvantage of needing to weigh the aggregates before they are heated and dried; hence, the knowledge of moisture content of the material becomes a key factor to ensure a good production [35]. In general, the consistency of materials going into a plant is a prerogative factor to maintain the asphalt production with minimum changes and delays [17].

### 5.1. How to Feed RAP Material into the Asphalt Plants

The traditional asphalt plant can be modified to incorporate the recycled aggregates in the production system following one of these three modes:

- adding cold RAP material at some stage of the asphalt production;
- pre-heating RAP material in a separate dryer;
- using a combined dryer to heat the RAP and virgin materials at the same time.

The first and second methods can be applied to batch plants [22]. The third method is mainly used in continuous asphalt plants, but it can be used in both systems [22]. Each RAP incorporation mode allows the introduction of the recycled aggregates at different points of the production plant based on the type of plant itself. In Table 3, the RAP incorporation modes and points are listed.

The first method of RAP inclusion introduces the recycled aggregates at ambient temperature; then, the overheated virgin aggregates will transfer the heat, drying them [10,19,22,36]. In this case, the RAP aggregates can be introduced at three different stages of the asphalt production: boot of the hot elevators, weight hopper and mixing drum. The introduction of RAP aggregates at the boot of the hot elevator requires the lowest investment and does not result in emission problems, as the scavenger system in the batch tower pulls out the generated steam [10,22]. If the RAP aggregates will be further screened, this procedure limits the maximum RAP content to relatively low percentages (10–15%) to avoid that the warm RAP aggregates clog the screens due to the consequent softening of the aged binder [10,22]. While, conveying the overheated virgin aggregates and the cold RAP material directly to the hot bins without passing through the screens allows the increase of RAP content up to 40% wt. [10]. The drawback of this procedure is the lower control on aggregates gradation. The second option incorporates the cold RAP aggregates into the weigh hopper between the release of two different hot bins of virgin aggregates [10]. This allows the RAP aggregates to be sandwiched between two layers of superheated aggregates, increasing the time to heat up the material itself [10]. In this case, no steam is generated and released, but an instantaneously thermal explosion will occur when the unmixed aggregates are discharged into the mixing drum; thus, to remove the steam generation, a baghouse with considerable capacity is required [10]. This solution permits the introduction of a higher percentage of RAP into the new formulations; however, the maximum RAP content is still limited to 25%. Thanks to an extra weigh hopper, the RAP material can be fed into the mixing drum over 20 to 30 s and this represents the third solution [10]. This option allows the production of recycled mixes with RAP content lower than 40% wt. [22,36]. The use of a specific feeder for RAP material permits a relatively better control on the generated steam by slowing down the batch cycle thanks to a control system; in detail, the system can extend the time of RAP injection into the mixer, giving more time to remove steam to the scavenging system [10,13]. Factors that control the amount of RAP feeding the batch plants are heating temperature for raw aggregates, RAP moisture content, temperature of the stockpiled RAP, final temperature of the bituminous mixture, production rate, exhaust capacity of mixing drum and percentage of fine particles in the RAP [10].

The second method requires a separate dryer to pre-heat the RAP material at a lower temperature than virgin aggregates, allowing the introduction of RAP material over 70% by the total weight of the mix [36]. This incorporation mode requires the upgrade of the plant, as the RAP material has to be conveyed to a separate hot storage bin equipped with its own weigh hopper [10]. Thus, the drawbacks of this method include the very high initial investment [10,22]. Furthermore, the superheating of virgin aggregates, albeit to lower temperatures, is still required [22].

The production of RAP mixtures in a continuous plant permits the introduction of higher percentages of recycled aggregates if compared to batch plants, as the RAP material is pre-heated. However, it is not possible to exceed a RAP content of 50% wt.; otherwise, the mixing plants could generate higher emissions, due to contact between recycled aggregates and the burner flame [10]. The factors that contribute to the emission problems are mainly moisture content of aggregates, amount of fines in RAP material and the relatively long time of exposure of the aged binder to the generated hot steam [10]. Regarding the application of the third method in continuous plants, a common technology is the parallel flow drum mixer, which allows the introduction of RAP aggregates at the halfway point. This solution foresees the introduction of virgin aggregates at the front of the heating drum (close to the burner), which form a dense veil of falling material that protects the RAP aggregates to a direct contact with burner flame [10,22]. In this solution, the exhaust gases and aggregates move in the same direction [10,22]. Changing the dryer configuration in a counter-flow drum mixer, where hot gases and aggregates move to opposite directions, it is possible to reduce the excessive gas temperature [10]. Hence, this system improves the environmental performance of the plant [22]. In a counter-flow drum mixer, a heating drum and a continuous mixing drum are combined in one unit allowing the heating and drying of virgin aggregates convectively and of the recycled material conductively [10]. In this system, the RAP material is usually introduced behind the flame, but multiple modifications are available on the market [10]. For continuous plants, the most advanced dryer drum is the double barrel dryer drum mixer. This system is an ordinary drum mixer with parallel- or counter-flow surrounded by a fixed outer dryer drum, called recycling ring [22]. The virgin aggregates are overheated in the inner drum; meanwhile, the RAP material is heated in the annular space (recycling ring) thanks to the energy transfer through the rotating shell. In this system, both virgin and recycled materials are not exposed to the hot gases or to the steam of the drying process; thus, the light fractions of binder are not removed from the material [10]. Moreover, the moisture removal from RAP aggregates allows a lower oxidation of the RAP binder in the mixing chamber when virgin binder and aggregates are mixed in the recycling ring [10]. This technology can be also used in batch plants. In this case, the entire system is a dryer drum and the recycling ring is used for drying and heating the RAP aggregates only. The holes through which the virgin aggregates are directed into the outer drum are also responsible for channelizing any smoke from the inner mixing section to the outer space [10]. The pollutants go directly to the flame where they are burnt. This results in a reduced hydrocarbon emissions [10]. The counter-flow dryer design also leads to higher production rates, with much lower fuel consumption [10].

### 5.2. Latest Advances in Asphalt Plants

Currently, there are different technologies that exploit the flexible design of batch plants to combine some of the aforementioned solutions in order to increase the RAP content in the final asphalt concrete. On the other hand, some ready-to-use technical systems that combine high RAP content with low emissions are also available. The asphalt plant manufacturers have developed innovative technologies to produce recycled asphalt mixtures with RAP content higher than 90% wt. [37,38]. In order to comply with the national environmental regulations in terms of emission, all asphalt plants exploit the advantages of the counter-flow system [37,38]. The novel technologies that have been recently developed differ for the type of heating drum or dryer drum. For instance, some technologies use a recycling drum with an hot-gas generator, which indirectly heat and dry the recycled aggregates [38], while others use two dryers. The virgin aggregates are heated and dried in the first dryer drum, while the RAP is heated in the second one thanks to the hot gases transferred from the first dryer [37]. This system prevents RAP material to come into contact with the burner flame and allows a more efficient system [37]. Thus, the production of recycled asphalt mixes with a RAP content towards 100% is becoming a feasible reality.

## 6. Mix Design of Mixtures with High and Very High-Content of RAP

Regardless the content of recycled material, the RAP asphalt mixtures should meet the same requirements of traditional asphalt concretes produced with virgin aggregates [15,21,25,26]. This means that the mix design must focus on the achievement of the required volumetric and mechanical properties to ensure good performances depending on the specific application [17]. Although the final goal of the mix design process does not change, the current procedures should be adapted considering the introduction of recycled aggregates covered with aged binder.

The well-known Superpave method for designing asphalt mixtures with RAP aggregates is very similar to the original one. The process differs in terms of [39]:

- Heating the RAP material more gradually and at lower temperatures compared to the virgin aggregates;
- Estimating the RAP aggregates density;
- Accounting for the RAP binder in the aggregates batching, since part of the RAP weight is aged binder;
- Reducing the virgin binder content to consider for the RAP binder;
- Possibly using a lower virgin binder grade or recycling agents to account for the RAP binder aging.

These differences can be found in each mix design method adopted worldwide, albeit with some distinctions. For instance, the American guidelines and standards refer to the specific gravity of all materials. Meanwhile, the parameter used in Europe, South Africa and Australia is the materials’ density. Moreover, the properties of a bituminous binder are evaluated differently in various countries. The Superpave method bases the characterization of binders on the definition of the Performance Grade (PG). The South African and European best practices define the binders using the penetration, softening point and viscosity. Recently, the Austroads association proposed a new design method based on the evaluation of the complex viscosity of blended binder to choose the virgin binder grade [15]. The definition of these parameters has a key role on mix design procedure and the prediction of the final performances of the asphalt concretes may be influenced by the type of the parameter that has been chosen by the designer.

Once the RAP material has been characterized from geometrical and physical point of view, the aged binder should be extracted and recovered in order to analyse the aggregates from RAP material according to the gradation and quality of typical HMA mixes [17]. Among all physical, geometrical and mineralogical properties of aggregates, bulk density is fundamental for the volumetric mix design and to determine the air voids present in new products [17,21]. The aged binder should be preferably removed by ignition test or solvent extraction; however, both methods may affect the gradation and geometrical properties of the recovered aggregates, reducing the accuracy of the results [17]. The ignition test can affect the physical characteristics of the RAP aggregates, while the solvent extraction cannot always remove all of the binder from the pores of the aggregates, leading to some errors in the measurement of the bulk density [17]. This inaccuracy does not represent a problem for asphalt concretes with low RAP content, but may become a significant issue in high-content RAP mixtures [17]. However, there are conflicting opinions on this topic, as more recent studies claimed that both methods do not appreciably affect the gradation and the geometrical properties of RAP aggregates and, consequently, the mix design of new formulations that contain RAP [21]. The recycled aggregates without aged binder (i.e., post-extraction), should be evaluated separately from virgin materials; then, the properties of the combined aggregates should be calculated using a blending formula to verify the acceptability of the entire material [17].

The procedure to select the virgin binder grade is strictly related to the RAP content. The Superpave method is based on a three-tier process where the limits of RAP quantity are 15% and 25% by weight. In detail, no changes in the mix design process are required with RAP content up to 15%; from 15% to 25% RAP, the virgin binder grade is lowered by one grade on both high and low temperature ends of PG. When more than the 25% RAP is considered, the selection of virgin binder is based on the use of blending charts [17]. This suggested method is settled on the percent of RAP material into the new asphalt mixes; however, it may be recommended to consider the RAP binder content instead of the quantity of the recycled aggregates, because not every RAP source has the same binder content [17]. Consequently, the decision of whether or not to characterize the RAP binder could be made according to the amount of binder replacement, rather than the common procedure [17]. The standard practice in South Africa supports this procedure, as the total RAP content should be determined by limitations imposed on the amount of aged binder in RAP material as a portion of the total binder content [3]. The limitations will be determined by virgin binder properties, as well as the final binder requirements, to meet performance criteria [3]. The South African guidelines specify the recommendations for mix design of RAP asphalt mixtures with RAP binder replacement up to 60%. SABITA suggests to use the blending charts for asphalt mix with RAP binder replacement greater than 15% wt. for determining both the softer binder grade and the rejuvenator dosage [3]. The European standard requires the properties of the blended binder if the RAP content exceed 10% wt. in surface courses or 20% wt. in binder and base layers [26]. All the current mix design methods for RAP mixtures consider a full blending between aged and virgin binders; thus, the additional dosage of virgin binder assumes the total activation of the aged binder [2,16,27]. However, this assumption is not correct as the blending falls between two extreme scenarios, typically described as full blending and black rock [40]. How much blending occurs between the RAP binder and added virgin binder is still debated [21,28]. A correct evaluation is fundamental since the assumption of full blending, i.e., the full activation of aged binder, will result in underestimated binder content of the resulting mixture if the RAP aggregates are partially behaving as a black rock [29], leading to cracking, ravelling and moisture damage of new formulations [19]. On the other hand, the assumption of black rock behaviour when RAP binder clearly contributes to the mixture performance will lead to a higher final binder content [29], which can cause plastic deformations of final asphalt concrete [19]. The addition of any recycling agent will change the blending between aged and virgin binders. However, it is still misunderstood how much the recycling agent affects the aged binder re-activation and its blending with the virgin one [28].

Following the terminology proposed by Lo Presti et al. [28], the blending of binders and the selection of recycling agents will be detailed in the following sub-paragraphs.

### 6.1. Bitumen Blending and Effect of Recycling Agents

During the service life of road pavements, binder undergoes aging and aggregates are progressively damaged [30]. The aging phenomenon influences the chemical composition of RAP binder, which leads to physical changes. In particular, the increase in asphaltenes proportion over the maltene fraction tends to enhance the stiffness and the viscosity of the RAP binder and to decrease its ductility [29,30]. In addition, processing, design and manufacturing procedures, together with the variability of RAP aggregates, affect the blending between RAP binder, virgin binder and/or recycling agents [28]. Thus, it is difficult to define the blending parameters for this complex material.

Each type of RAP has a minimum amount of aged binder that will be available or active under certain production conditions, i.e., time and temperature; this is an intrinsic property of the recycled material defined as degree of binder activity (DoA) [28]. It is important to underline that the degree of blending between RAP and virgin binders is a function of production parameters as shown by the study of Mogawer et al. [31]. The portion of unavailable RAP binder that will not be activated during production process consists of two components: black rock RAP and absorbed RAP binders [28]. The former is a very stiff and brittle aged binder that is considered as a part of RAP aggregates, while the latter is the portion of binder that has been absorbed by RAP aggregates and does not act in the binder film [28]. Thanks to the addition of virgin binder that can be softer than usual, it is expected that the RAP binder attains a necessary viscosity and it blends with the virgin binder to constitute a uniform coating on the aggregates during mixing [19]. The virgin binder should sufficiently diffuse and blend with the aged binder to recover its properties [41]. In this scenario, the degree of binder activity corresponds to the degree of binder availability (DoAv) [28] and this quantity has to be considered by designers to take into account the total amount of binder in the final asphalt concrete. The DoAv should increase if a recycling agent is added to the mixture. The recycling agents aim to lower the viscosity of RAP binder improving the workability of the mix and to recover the RAP binder properties. In particular, the study of Carpenter and Wolosick summarized their diffusion into hardened binder in subsequent steps [42]:

- The recycling agent forms a very low-viscosity layer on the film of aged binder;
- The agent begins to penetrate the RAP binder and its amount over the aggregate decreases in time leading to soften the aged binder film;
- After a specific period, no recycling agent covers the aggregate and, simultaneously, dividing the coating layer of binder in two parts, the viscosity of the inner layer is lowered and the viscosity of the outer layer of binder is increased until the equilibrium is approached.

Thanks to the softening effect of the recycling agents, the quantity of black rock RAP binder, and, eventually, of the absorbed RAP binder can be lowered [28]. In this second scenario, the DoAv consists of active RAP binder (DoA), activated RAP binder and the residual amount of recycling agent [28]. As described, the diffusion process occurs over a certain period of time; thus, designers should be aware that the DoAv may change at different stages of production and the time between mixing and subsequent testing of mixture is critical [28,42]. Different recycling agents in different concentrations may diffuse at different rates; moreover, the characteristics of aged RAP binder may affect the diffusion process [28,42]. The addition of diluent oil fractions or the rise in the production temperature may increase the diffusion rate because the process depends on temperature, based on the Arrhenius equation [43]. As for the addition of oily fractions, it was found that molecular weight and, to a larger extent, molecular shape affect the diffusion process and aliphatic compounds diffuse more rapidly than bulk condensed compounds [43]. The efficiency of blending with recycling agents is represented by the degree of blending (DoB) parameter, which is an indicator of the contribution of the aged binder and the recycling agents on the final blended binder [28]. Several studies proposed the evaluation of DoB by the use of mechanical, rheological and chemical measurements at the binder level; however, there is a lack of standardized procedures to evaluate all blending parameters [44].

### 6.2. Recycling Agents Selection and Dosage

A preliminary distinction should be made among recycling agents that include softening and rejuvenating agents. The softening agents or lubricants are able to lower the viscosity of the aged bitumen [30]; they consist of asphalt flux oil, lube stock, lubricating or crankcase oil and slurry oil [45]. On the other hand, the rejuvenators have the capacity to restore, even if partially, the chemical and physical properties of aged binders, re-balancing the composition of the hardened RAP binder [30]. They often consist of lube extract (fraction removed from lube stock) and extender oils, which are rich in naphthenic or aromatic fractions [45]. The decision whether to use softening or rejuvenating agents is taken in order to meet the requirements of the new formulations and the designing conditions, based on the quality and the stiffness of the RAP binder, the RAP content, the amount of virgin binder to be added, the characteristics of the final mixture, etc. [46].

The recycling agents are designed balancing, on one hand, the stiffness reduction of RAP mixtures improving their workability, and, on the other hand, the increase of the effectively available RAP binder, avoiding the over-softening of the mix, which can cause rutting problems, bleeding and insufficient stability [30,47]. Correct rejuvenators should be selected in order to provide adequate short- and long-term properties of the total binder and the new asphalt concrete. In the short term, the rejuvenators should allow the production of high and very high-content RAP mixtures, thanks to a rapid diffusion into the aged binder and its mobilization, in order to cover the aggregates uniformly and to produce a workable mixture without the generation of harmful emissions [14]. In the long-term, rejuvenators should reconstitute the chemical and physical properties of the aged binder for a new service period [14]. The rheology of the blended binder has to be altered to reduce cracking susceptibility without over-softening the binder and guarantee a sufficient adhesion and cohesion with the aggregates [14]. Moreover, the rejuvenators should ensure stability and durability of the blended binder [30]. The stability is achieved when the asphaltene are well peptized [30] and the high content of polar and aromatics of rejuvenators was discovered to enhance the dispersion of asphaltenes [48]. On the other hand, products with a high percentage of saturates were found to be detrimental on hardening susceptibility and ductility of the blended binder [49]. In general, a recycling agent made from the same crude oil of the aged binder is probably highly compatible and the RAP binder is more likely optimized [30]. Additionally, ongoing studies on bio-based recycling agents are obtaining promising results [33,50].

The dose of recycling agents should be selected to meet the target PG or basic properties (i.e., penetration, softening point and viscosity) of the blended binder according to the American or European/Australian/South African regulations, respectively. This should lead to improve the cracking resistance without adversely affecting the rutting potential of the resulting RAP mixture. A study of Zaumanis et al. [33] has compared various rejuvenators type and dosages. They found an almost linear increment of the Superpave PG at both high and low temperatures of the blended binder varying the added dose of the same recycling agent, while the penetration values increase exponentially in the same conditions [33]. Furthermore, the softening effect of organic products was found to be higher if compared to the one of some petroleum-based agents [33]. The mixing of the recovered RAP binder with recycling agent to determine the rejuvenated binder characteristics is considered the best approach [14]. Among the proposed methods to quantify the blending parameters, various studies have demonstrated that chemical investigations can help to the definition of recycling agents effects, as they can distinguish between softening agents and rejuvenators, defining the best responses of blended binders between several good rheological behaviours [51].

The use of incompatible recycling agents or overdosage can cause a lack of binder cohesion and reduce adhesion with the aggregate, which turn into premature pavement deterioration and higher water sensitivity [14]. Field studies have demonstrated that the use of incompatible products or excessive amount of recycling agents result in the migration of oily fractions to the surface of the asphalt layer, which results in the reduction of the friction of wearing course layers and compromised pavement performance [14].

The effectiveness of a rejuvenator depends on the uniform dispersion of the product within the recycled mixture and its diffusion into the aged binder coated outside of the aggregates [32]. In asphalt plants, the recycling agents can be added in various points, but it is essentially sprayed on the RAP aggregates or pre-blended with the virgin binder [32]. Each dosing point presents advantages and disadvantages; the EAPA association suggests adding the rejuvenator to the RAP material at the discharging point of the parallel drum [46]. This position has two advantageous aspects: the recycled aggregates are already pre-heated and, after the addition of the rejuvenator, the RAP will be stored for some time at the reclaimed asphalt silo prior to mixing [46]. Both aspects facilitate the diffusion of the rejuvenator in the RAP material. However, the present solution is only possible when the recycling agents have a suitable flash point temperature and thermal stability [46].

## 7. Mechanical Behaviour of Asphalt Mixtures Containing RAP

At low RAP contents, the properties of asphalt concretes with RAP are not significantly different if compared to mixes without recycled aggregates; thus, the presence of RAP aggregates can be considered as negligible [52]. The NCHRP project D9-12 found the threshold value to fall between 10% and 20% of RAP content, depending on the stiffness of aged binder [52]. However, at higher RAP contents, the mechanical behaviour of RAP asphalt mixes generally reveals an increased stiffness, which could lead to cracking phenomena if no adjustments in the mix design are made. Conversely, this type of mixture would exhibit more resistance against rutting [52], but this advantage can be reduced or nullified by the incorrect use of recycling agents [14]. The water sensitivity of HMA with high RAP content is normally lower if compared to virgin mixes; however, this characteristic can be affected by many factors as previously mentioned. As a consequence, the correct mix design with performance-related tests can help the evaluation of the mixture properties [19]. The performances of high and very high-content RAP mixes are reported and compared in this section, with a specific focus on potential distresses associated to cracking and rutting phenomena and water susceptibility.

### 7.1. Performances of Recycled Mixtures with High RAP Content

The aged binder present in the RAP leads to increase the stiffness of the final asphalt product [23]. This typical response can be found in several laboratory studies. An Australian experimental program analysed the air void content and the resilient modulus (AS 2891 standard) of hot dense asphalt mixes containing 0%, 10%, 20% and 30% of RAP from the same source [23]. Virgin and 10% RAP mixes were produced with standard bitumen; the recycled asphalt mixes with RAP content greater than 15% were made with a softer binder, according to the recommended practices of Victoria state at that time [23]. Despite that all mixtures were designed to have similar gradation and acceptable coating of aggregates by the resulting blended binder, the results indicated that the recycled aggregates influenced both the volumetric and stiffness properties of the samples [23]. In detail, the increase in RAP content led to an enhancement in the stiffness of the mix and reduced the air voids due to the higher content of the resulting blended binder [23]. The researchers attributed the variations to the differences in fines proportion, binder viscosity and the level of blending between old and new binders [23]. A direct proportion between RAP content and stiffness of RAP mixtures was also found during the NCHRP project 09-46 [21]. This study involved the analysis of two sets of asphalt mixtures with different nominal maximum aggregate sizes, which incorporated various RAP amounts ranging from 0% to 55% by weight of the aggregates [21]. The experimental plan used a variety of materials from four different locations in the USA, and the results of dynamic modulus (AASHTO TP 62-07 standard) of RAP asphalt mixes were significantly influenced by the RAP content and the source of recycled aggregates [21]. Conversely to these findings, other researchers did not find a direct proportion between RAP content and mixture stiffness. A Latvian study and an Australian project underlined that low RAP content did not lead to an increase in this property dramatically as high proportion of RAP, and the RAP mixture stiffness can even be lowered, changing the grade of the used virgin binder. The Latvian study compared four mixtures: a reference mixture with virgin materials, two 30% wt. RAP asphalt mixes produced with two RAP sources (unprocessed and processed) and a 50% wt. RAP mix made with unprocessed RAP. The virgin asphalt concrete was produced with a 50/70 pen bitumen, while for every recycled mixture, a 70/100 pen bitumen was adopted [53]. The results of both 30% RAP mixtures showed lower stiffness (standard EN 12697-26, Annex B) than that of the reference mix, which showed similar stiffness of recycled asphalt concrete with the highest RAP content (50% wt.) [53]. Thus, the use of softer binder allowed the mitigation of the stiffening effect of RAP aggregates when they were added up to 30%. Similar results have been found during the Austroad project (Australia) that compared different dense graded asphalt mixtures with RAP content up to 60% wt. without founding a statistically significant increment of flexural stiffness (EN 12697-26 standard) of RAP mixtures up to 30%, regardless the penetration of the virgin bitumen [15]. All asphalt mixes were designed to keep the aggregates gradation and total binder content unaltered. Hence, based on the quantity of RAP binder, a specific amount of virgin binder was added to meet the final quantity evaluated during the mix design [15]. The introduction of RAP material stiffened the resulting RAP mixes; however, the performances of the asphalt mixtures with RAP content up to 30% wt. using bitumens with different grade confirmed that the use of softer virgin binder can correct the final response of the mix [15]. The limited literature review confirmed that the stiffness of high RAP mixtures is generally greater than the corresponding virgin mixes. This behaviour might be beneficial for specific design purposes (i.e., perpetual pavements and high modulus asphalt concrete layers) [14] while, for conventional asphalt mixtures, the stiffness has to be reduced to avoid potential cracking phenomena [25,31]. This possible drawback was investigated in the abovementioned studies in terms of fatigue cracking resistance, showing a reduced fatigue life of RAP asphalt mixes [15,21,53]. In addition, the NCHRP project investigated the potential low-temperature susceptibility of the RAP asphalt concretes. Even though a clear trend was found with regard to fatigue behaviour of RAP asphalt mixtures, the thermal cracking showed conflicting results in terms of the Semi-Circular Bending (SCB) test, but high content RAP mixes performed similarly to the corresponding virgin ones by the use of the Bending Beam Rheometer (BBR) test [21]. The Latvian studies evaluated the fatigue resistance following the EN 12697-24 standard, Annex D [53]. The RAP mixture with 50% wt. of recycled aggregates was more prone to fatigue cracking, even if it had a similar stiffness of the virgin mix [53]. Thus, the softer binder mitigated the stiffness of this recycled mixture without improving its fatigue life [53]. As mentioned before, the combination of higher stiffness and brittleness will likely result in a shorter fatigue life and higher probability of thermal cracking occurring in the field [53]. A comparison between the use of standard and softer binder does not provide a clear and expected increase in fatigue resistance due to the presence of the softer binder. In detail, during the Australian project, the resistance against fatigue cracking was evaluated following the Austroads AGPT/T233 standard and the results show a reduction of fatigue life increasing the percentage of RAP aggregates for mixtures made with softer binder [15]. Moreover, 30% RAP mix with softer binder had the same performance of mixes without RAP material [15]. Meanwhile, a less clear trend was observed of RAP mixtures with standard bitumen [15].

For what the permanent deformations is concerned, all studies found that recycled asphalt mixtures had acceptable or better rutting resistance compared to the virgin mixes [15,21,53]. The American project NCHRP 09–46 evaluated the resistance against permanent deformations by the use of the Flow number test in confined configuration, and none of the samples exhibited tertiary deformations [21]. It was found that rutting sensitivity mainly depends on the source of materials and high PG of virgin bitumen, rather than RAP content [21]. In addition, the moisture susceptibility of all asphalt concretes was evaluated in accordance with the AASHTO T 283 standard [21]. The presence of recycled aggregates improved the responses of asphalt mixes, even if several recycled mixtures did not meet the requirements [21]. The Australian and Latvian studies used the wheel tracking test to evaluate the rutting potential of asphalt mixtures according to European (EN 12697-24, Annex D) and Australian (AGPT/T231) standards. In the Latvian study, the largest average rut depth appears for the reference mixtures (without RAP material); however, the authors ascribed this behaviour to the highest bitumen content [53]. The RAP mixture with 30% wt. of RAP, which was processed, showed the highest resistance [53]. In general, the deformation susceptibility was acceptable for all recycled mixtures, meaning that RAP content had no significant effect on rut resistance [53]. The Australian results showed a reduction of rut depth when RAP content increased [15]. The use of softer binder in recycled asphalt mixes led to a decrease of the rutting resistance of 15% RAP mixes when compared to the same mixture made with standard bitumen [15]. Furthermore, all mixtures did not present issues in terms of moisture sensitivity (AGPT/T232 standard) [15].

Mogawer et al. conducted a similar laboratory study on recycled HMA materials with the aim to evaluate the effect of both production processes and type of components on the performances of the final asphalt concretes [31]. Mixtures with different nominal maximum aggregate size and two virgin bitumens (standard and soft) were compared, containing recycled aggregates in various proportions from 0% wt. to 40% wt. [31]. The samples were manufactured in three different asphalt plants located in Vermont, New York and New Hampshire (USA) [31]. The study revealed an enhanced mixture stiffness (AASHTO TP62 standard) when the RAP content was above 30%. On the contrary, the 20% RAP mixture had lower stiffness than the control mix with virgin materials [31]. The evaluation of cracking resistance confirmed the stiffness data: higher RAP content resulted in a lower fatigue life at moderate to high levels of deflection according to the Overlay Tester (Tex-248-F standard) [31]. The use of soft bitumen mitigated the stiffening effect of recycled aggregates and hardened RAP binder, but the PG grade of virgin bitumen was two grades softer to improve the cracking resistance of recycled mixtures [31]. The Hamburg Wheel-Track Testing (AASHTO T324 standard) was used to estimate the rutting and moisture resistances of all mixes. Only the HMA mixtures from one state showed higher moisture susceptibility regardless of bitumen type, amount of RAP or production parameters. This might be ascribed to the poor quality of materials, since uncoated fine materials were released from the samples during the test [31]. On the other hand, RAP mixtures showed enhanced rutting resistance, which improved as the amount of RAP in the mix increased [31]. The use of softer bitumen did not consistently or significantly affect the rutting potential and moisture susceptibility of the mixtures [31]. Generally, plant production and RAP storage practices, as well as the handling of materials prior to specimen fabrication (i.e., reheating loose mix or not), affect the mixture performances [31]. Furthermore, the stiffness data indicate that longer storage times of the mix may nullify the possible benefit of the use of softer binder [31].

In general, the laboratory studies showed an increased stiffness of recycled asphalt concretes, which turns into lower fatigue life and improved rutting resistance of the products. This trend depends on the RAP content, but some researchers proved that it is less evident when RAP content lower than 30% wt. is incorporated within the mixture, which confirms the conclusions of the NCHRP project D9-12. Furthermore, the use of a softer virgin bitumen may mitigate the stiffening effect of the residual aged binder. On the other hand, the incorporation of recycled aggregates does not cause significant negative effects on the resulting RAP asphalt mixtures. However, the production process has a crucial impact on the final performance of the RAP asphalt mixes [31].

### 7.2. The Effect of Recycling Agents in Very High-Content RAP Mixtures

Very high RAP content or 100% recycled asphalt mixtures may meet the standard requirements by adding recycling agents. So far, the studies evaluated the effects of different types of agents.

Porot et al. investigated the performance of HMA mixtures containing very high content of RAP material (70% wt.) with and without the introduction of a bio-based recycling agent, which were compared with a reference formulation made only with virgin materials [50]. All asphalt concretes were designed with a similar gradation and made with a 40/60 pen bitumen [50]. The chosen recycling agent allowed the reduction of the stiffness modulus (EN 12697-26 standard) of the RAP mixes caused by the introduction of recycled aggregates [50]. Thanks to the construction of master curves, the authors compared the responses of RAP mixtures with agent and the reference mix in different service conditions. The use of the recycling agent in RAP mix did not enhance the rutting potential of the mixture at high temperature (40–60 °C), keeping the benefit of RAP against rutting resistance. At the same time, it improved the response of the asphalt concrete at low temperature (below 0 °C) indicating that the mix was less prone to cracking [50]. The behaviour at low temperature was confirmed by the evaluation of cracking susceptibility by means of the Thermal Stress Restrained Specimen Test (TSRST) on prismatic samples (EN 12697-46 standard) [50].

Other studies that were focused on the characterization of totally recycled asphalt mixtures evaluated the same softening effect of recycling agents regardless their origin. The first considered research compared the stiffness of 100% RAP asphalt mixture with the addition of a commercial product made with various chemicals and waste engine oil [54], while the second one focused on a bio-based agent (distilled tall oil) comparing two different sources of RAP aggregates [55]. Both studies evaluated the stiffness modulus of the asphalt concretes following the EN 12697-26 standard. In the first study, the commercial product and the waste engine oil guaranteed a higher prevalence of the viscous component of the stiffness modulus at high test temperature, improving the flexibility of the totally RAP mixes; among recycling agents, the waste engine oil was slightly more efficient than the commercial agent [54]. The second abovementioned study highlighted the positive effect of the introduction of the bio-based rejuvenator, which reduced the stiffness of the aged recycled asphalt mixes [55]. In terms of fatigue resistance, the RAP mixtures manufactured with recycling agents, especially with the commercial product, showed the best fatigue resistance (EN 12697-24 standard) [54]. On the other hand, the bio-based agent allowed the increase of fracture toughness of the RAP mixes (EN 12697-44 standard). However, the authors affirmed that the test temperature may not be sensitive enough towards mix design parameters influencing the obtained results [55]. The rutting resistance of the RAP mixtures of both studies was not negatively affected by the addition of the recycling agents; this characteristic was defined according to the EN 12697-22 and EN 12697-2 standards, respectively [54,55]. However, the recycled mixes without recycling agents had better resistance and the commercial product performed better in terms of restoring the RAP asphalt mixtures properties [54]. Thanks to the comparison of different RAP sources and blended binder content, the resistance against permanent deformations was improved by using a coarser gradation and lower binder content. In addition, the study of Silva et al [54], evaluated the water sensitivity of asphalt mixtures (EN 12697-12), finding good results, although the mix without recycling agent was slightly more sensitive to water [54]. With reference to moisture susceptibility, the waste engine oil had better performance compared to the commercial product [54].

All in all, the recycling agents can effectively allow the reduction of stiffness and the potential cracking distresses of the final RAP mixes. However, their introduction should be properly designed in order to avoid any negative effects with regard to water sensitivity and rutting phenomena.

All the data collected from the literature review are reported in Table 4 in order to provide an overview of the state-of-the-art on the use of RAP mixtures. Novel production technologies, adequate RAP management, improved mix design and advances in performance-related test methods, especially focused on the cracking phenomenon, will improve the confidence in the use of very high-content or even 100% RAP asphalt concretes, allowing also the development of performance-based specifications [14]. The use of proper engineering evaluations of the asphalt mixtures helps the use of RAP material at its fullest extent in any type of bituminous mixtures [17].

## 8. Environmental and Economic Aspects

The use of recycled materials in asphalt pavements preserves non-renewable resources, reducing the extraction of aggregates and the use of fresh bitumen, decreasing the amount of material landfilled [8,56,57]. Moreover, it reduces the overall asphalt mixture costs [8].

As previously mentioned, in 2018 the United States disposed a very low percentage of RAP materials by the total amount that was declared by producers. It means that the RAP materials accepted by USA asphalt plants saved about 46.7 million cubic meters of landfill space in one year only [8]. However, in order to have a complete assessment of the environmental benefits given by the incorporation of recycled materials in new HMAs, the air emissions and energy consumption during the entire life cycle of road infrastructures should be considered and evaluated. The asphalt mixtures production has its own carbon footprint, that is, the total amount of produced greenhouse gases (GHG) [22]. Regardless the use of RAP aggregates, the production of asphalt mixtures mainly releases carbon dioxide (CO_2_), methane (CH_4_) and nitrous oxide (N_2_O) that comes from the production of raw materials and asphalt mixtures, transportation, paving and rolling of the asphalt mixtures [58]. There are different sources of GHG emissions during the entire production process, mainly generated by the energy consumption of machineries and plants [58]. The production of raw materials includes rock blasting, quarrying, hauling, crushing and screening for production of aggregates, while the production of bituminous binder involves crude oil extraction, transport and refining [58]. In the plant, fossil fuel is consumed for the heating of components and during the mixing phase. The engines of the transport vehicles, pavers and rollers release air emissions during the transportation, laying and compaction phases. Moreover, the GHG emissions from hot mix asphalt should be considered [58]. Usually the GHG emissions are expressed in CO_2_ equivalent because the more energy is required for a product, the more fuel is burnt and the more carbon dioxide is released [22]. A case study in China reveals that 54.01% of GHG emissions in CO_2_ equivalent are released during the production of asphalt mixture and 43.18% are generated during the raw materials production phase [58]. The transportation of both raw materials and asphalt mixes produces about 1.35% of the total CO_2_ equivalent, while, 0.86% and 0.61% of the total GHG emissions are due to the laying and compacting phases, respectively [58]. The use of RAP aggregates allows the reduction of CO_2_ equivalent released by the raw materials productions, which is one of the biggest contributors of GHG emissions [58]. Excluding the raw materials production, most CO_2_ equivalent emissions are caused by the production of asphalt mixture, and the dryer drum has a leading role on the carbon footprint of the plant [22,58,59]. The incorporation of RAP aggregates at ambient temperature leads to more fuel consumption to dry the materials, since the current RAP recycling technologies require virgin aggregates to be superheated, even if a separate dryer drum is used for RAP [22,57]. The superheating temperature varies with the type of technology used and depends on moisture content of RAP aggregates, mix discharge temperature and RAP content [22]. In general, the batch plants show higher GHG emissions than those of continuous plants [57]. Higher amounts of RAP on the total mass of the mix require higher temperatures to superheat the virgin aggregates, and thus more energy, but this energy consumption is partially compensated by the lower mass of the virgin aggregates [22]. Considering the theoretical amount of energy needed to be transferred to aggregates, the carbon emissions released by specific RAP technology can be evaluated by the use of a conversion factor. In detail, considering a preheating temperature and moisture content, the lowest consuming technique is the cold feed that includes 30% of RAP material, followed by counter-flow 50% RAP, pre heater 60% RAP, double barrel 50% RAP and parallel 50% RAP [22]. The maximum recommended RAP percentage for each specific technology, in most cases, will use less energy than the conventional production [22]. Pushing the use of RAP materials for the production of totally recycled asphalt mixtures would allow the reduction in CO_2_ of 18 kg and saves approximately 20% of energy per ton of paved mixture, according to the study of Zaumanis et al [14]. This evaluation does not consider the milling of the old pavement, since it was considered an integral part of reconstruction and would be done irrespective of the type of laid mixture, and it assumed the addition of recycling agent without the use of any virgin binder, as commonly done [14]. The tools based on the Life Cycle Assessment (LCA) approach allow the evaluation of the environmental impact of various asphalt mixtures, with and without RAP aggregates, considering the entire in-service life of the infrastructure and its disposal. The study of Vidal et al. [56], which investigated the LCA of pavements from construction to their end-of-life, confirms the crucial impact of raw materials production, together with the production of asphalt mix [56]. As highlighted before, the introduction of recycled aggregates reduces the GHG emissions related to raw materials production, but it additionally decreases the emissions at the end-of-life stage, since it avoids the asphalt landfilling [56]. In detail, the research reveals the beneficial effects of RAP material introduction on climate change and fossil sources depletion; the incorporation of 15% of RAP material leads to a reduction of the impacts on climate change by 13% and the impact on fossil sources depletion by 14% [56]. The cumulative energy demand highlights that the total energy for the asphalt mixtures mainly depends on the RAP content [56]. All the reported values were evaluated for a specific case study; hence, the uncertainty and the variability of the results should be considered.

**Figure 2:**
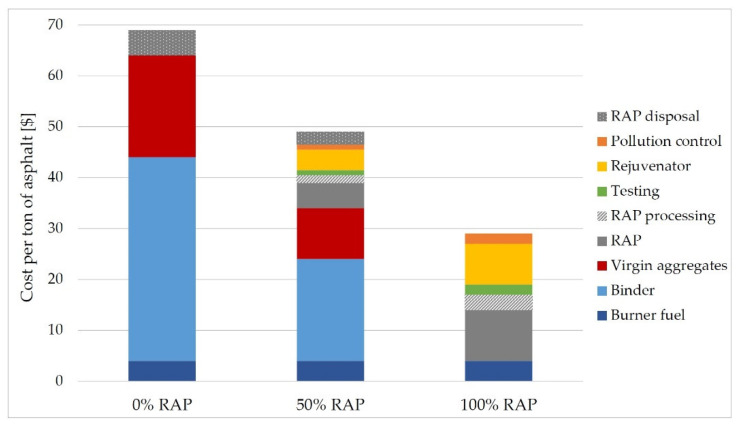
Recycled hot mix asphalt costs based on RAP content–Adapted from [14].

Beyond the environmental benefits, the use of RAP aggregates helps to save costs related to raw materials. The survey conducted by NAPA states that the USA saved 2.8 billion dollars in 2018 thanks to the replacement of virgin aggregates with recycled materials. This substitution reduced the need for 4.1 million tons of virgin binder and 78 million tons of virgin aggregates [8]. The savings were estimated considering the average price of both virgin materials and the additional cost realized by diverting RAP from landfills [8]. Among all factors, major savings can be ascribed to the replacement of virgin binder by the hardened binder [14,19]. These savings must be quantified to account for additional costs related to RAP processing, testing, use of recycling agents and the cost of purchasing RAP if the material is not enough, or RAP disposal if the amount is too large [14]. The use of very high content RAP mixes and totally recycled asphalt mixtures would require significant investments for the modification of the production technology that cannot be neglected [14,60]. Zaumanis et al. [14] calculated the cost of a ton of RAP asphalt mixture varying the RAP content and the results are approximatively represented in Figure 2. The cost analysis considered a RAP mixture with an aggregates content of 94.3% and blended binder content of 5.7% (RAP binder 5.1% and recycling agent 0.6%) and the costs per ton of 100% RAP mixture would be reduced between 50% and 70% if compared to virgin mix, depending on the market availability of RAP [14]. In particular, the costs related to the upgrading of the plant technologies greatly vary depending on the chosen technology and the availability of the equipment [14]. Taking into account the costs of plant upgrading, the profit margin is ranging from 0 to 40 dollars per ton of mix [14]. The profit per each ton of produced mixture will likely not be directly related to the savings as calculated because this depends on the quality and the durability of the 100% RAP pavement, and, unfortunately, the existing state-of-the-art technology does not allow for a conclusive evaluation of long-term performance of 100% RAP mixes [14].

## 9. Discussion and Conclusions

The present article reviews the use of high (over 40% wt.) and very high content (up to 100% wt.) of RAP in asphalt mixtures, highlighting the possible limitations and restrictions related to the introduction of high quantity of RAP aggregates. In detail, this review describes the use of RAP in different countries, summarizes the best practices in RAP management, presents the novel production technologies, defines the adaptation of the mix design procedures to take into account the incorporation of recycled aggregates coated by aged binder, reports the performances evaluated in various experimental studies and considers the economic and environmental benefits connected to the inclusion of RAP. Based on the present study, the following aspects can be highlighted:

- The RAP content limits specified by existing regulations and guidelines reflect the general misconception that considers the recycled aggregates and the corresponding asphalt mixes as low-value products compared to virgin ones. The correlated reasons can be mainly ascribed to the intrinsic higher variability of the raw material, the practical restrictions imposed by the technologies of the traditional asphalt plants and the natural increased stiffness of the final products, which may cause the reduction of fatigue and thermal cracking resistances. Lastly, missing protocols for the characterization of RAP components and an approved mix design do not encourage a greater use of this recycled material.
- The knowledge gap related to the characterization of the RAP binder and the blending phenomenon that occurs between recycling agents, virgin and aged binders is still present. Consisting of aggregates and aged bitumen, the properties of RAP material are affected by multiple variables that are strongly related to the aging experienced during the previous in-service life of pavements. The properties of the RAP material itself have to be considered for setting the production variables; in turn, the production parameters affect the behaviour of the RAP and the blending phenomenon while combined with the virgin materials. Hence, an extensive and complete characterization of the material is crucial to define a proper mix design and to predict the performances of the final mix.
- The general wisdom refers the increased stiffness of the RAP asphalt mixtures to the aged bituminous binders that coat the recycled aggregates. The use of soft binders and/or recycling agents can mitigate the stiffening effect of the RAP content, improving the workability of the mix and facilitating the blending between the aged and the virgin binders. However, the introduction of recycling/softening products represents an additional variable that has to be correctly designed in order to avoid detrimental effects in terms of the rutting resistance of the final products.
- The incorporation of RAP material in recycled asphalt mixtures can strongly affect the economic and environmental impacts of the construction of a road pavement. Hence, life cycle cost analysis investigations are considered important tools to select and design the construction materials for transportation infrastructures.
- The design and production of high and very high content RAP asphalt mixtures is more challenging than traditional ones. An adequate management of RAP that allows the reduction of the raw material variability, the use of modern technologies that permits a controlled and maximized introduction of the RAP aggregates and a performance-based methodology of design result in a more efficient RAP utilization.

## Figures and Tables

**Table 1:** Comparison of Reclaimed Asphalt Pavement (RAP) uses in the USA and Europe: data from [8,12].

Considered Quantities	USA	EUROPE
Total production of HMA and WMA	389.3 × 10^6^ Tons	297.9 × 10^6^ Tons
Total RAP accepted in facilities of plants	101.1 × 10^6^ Tons	49.5 × 10^6^ Tons
RAP used in HMA/WMA mixtures	81.3%	51.4%
RAP used in CMA mixtures	0.297%	3.81%
RAP used as aggregates for unbound layers	6.33%	17.0%
RAP used for other purposes	1.98%	2.00%
RAP landfilled	≈0.00%	9.63%

**Table 2:** Recommended geometric shape of the unprocessed and processed RAP stockpiles.

Critical Phenomenon	Unprocessed RAP	Processed RAP
Segregation	Arc-shaped and layered stockpiles [16]	Conical or low-sloped stockpiles [10,16]
Consolidation	Stockpiles with limited height [20]	
Moisture retention	Conical stockpiles—No irregular shape [20]	

**Table 3:** Overview of the RAP incorporation modes and points in batch and continuous plants.

RAP Incorporation Mode	RAP Incorporation Point	Type of Plant
Addition of cold RAP at some stage [10,22]	Boot of the hot elevator—Subsequent screening and storing in hot bins Boot of the hot elevator—Direct storing in hot bins Weight hopper Mixing drum (pugmill)	Batch
Pre-heat RAP in a separate dryer [36]	Separate dryer drum for RAP aggregates only	Batch
Pre-heat RAP and virgin aggregates in a combined dryer [10,22]	Half-point of the dryer/mixing drum with either parallel- or counter-flow Beyond the burner flame of the dryer/mixing drum Recycling ring (annular space) of the double barrel dryer drum mixer Recycling ring (annular space) of the dryer drum	Continuous
		Batch

**Table 4:** Performances of asphalt mixtures with low, high and very high content of RAP.

References	Type of Mix	Stiffness and Cracking	Moisture Damage	Permanent Deformations
Binh T. et al (2011) [23]	RAP content = 0, 10, 20, 30% Binders = soft and standard	Higher RAP contents lead to increase the stiffness of the mixes—no mitigation effect of soft binder		
West R. et al (2013) [21]	RAP content = 0, 25, 40, 55% Various materials	Higher RAP contents lead to increase the stiffness of the mixes—no use of soft binder Higher RAP contents turn into lower fatigue resistance of the mixes Not clear trend of thermal cracking	Several RAP mixes did not meet the standard criteria, but they show a better moisture resistance than virgin ones	Good results of all mixes
Lee J. et al (2015) [15]	RAP content = 0, 15, 30, 60% Binders = soft and standard	No significant differences in mixes with different RAP contents—mitigation effect of soft binder Higher RAP contents turn into lower fatigue resistance of the mixes—mitigation effect of soft binder	Good results of all mixes	Higher RAP contents turn into higher rutting resistance—soft binder increases the rutting potential of RAP mixes
Izaks R. et al (2015) [53]	RAP content = 0, 30, 50% Binders = soft and standard	The mix with the highest RAP content has similar stiffness of virgin mix—mitigation effect of soft binder Higher RAP contents turn into lower fatigue resistance of the mixes—mitigation effect of soft binder		Good results of all mixes—30% RAP mix has the better response
Mogawer W. et al (2012) [31]	RAP content = 0, 20, 30, 40% Binders = soft and standard	Higher RAP contents lead to increase the stiffness of the mixes Higher RAP contents turn into lower cracking resistance of the mixes	Higher RAP contents lead to increase the rutting resistance—importance of materials quality and binder coating—no significant effect of soft binder	Higher RAP contents turn into higher rutting resistance—importance of materials quality and binder coating—no significant effect of soft binder
Porot M. et al (2017) [50]	RAP content = 0, 70% Additional mix = 70%RAP + recycling agent One binder	Higher RAP contents lead to increase the stiffness of the mixes—mitigation effect of recycling agent		
Silva H.M.R.D. et al (2012) [54]	RAP content = 100% Additional mixes =100%RAP + recycling agents One binder	The mix with higher RAP content and no recycling agent has higher stiffness—mitigation effect of recycling agent The RAP mix with recycling agent has the best fatigue resistance—mitigation effect of recycling agents	Good results of all mixes—the RAP mixture without recycling agent is slightly more sensitive	Good results of all mixes—RAP mix without recycling agent is slightly better
Zaumanis M. et al (2019) [55]	RAP content = 100% Addition of recycling agent Binders = PmB and standard	The stiffness of all RAP mixes is similar to that of virgin one—mitigation effect of recycling agent Not clear trend of fracture toughness		The RAP mix with the lowest binder content and coarser gradation has the best rutting resistance
